# Investigation of molecular heterogeneity of β-thalassemia disorder in District Charsadda of Pakistan

**DOI:** 10.12669/pjms.322.9415

**Published:** 2016

**Authors:** Muhammad Shakeel, Muhammad Arif, Shoaib Ur Rehman, Tabassum Yaseen

**Affiliations:** 1 Dr. Muhammad Shakeel, PhD (UK). Department of Biotechnology, Bacha Khan University Charsadda, Khyber Pakhtunkhwa, Pakistan; 2 Mr. Muhammad Arif, M.Phil. Department of Biotechnology, Bacha Khan University Charsadda, Khyber Pakhtunkhwa, Pakistan; 3 Dr. Shoaib Ur Rehman, PhD (Pakistan). Department of Biotechnology, Bannu University of Science and Technology, Bannu, Khyber Pakhtunkhwa, Pakistan; 4 Dr. Tabassum Yaseen, PhD (Pakistan). Department of Botany, Bacha Khan University Charsadda, Khyber Pakhtunkhwa, Pakistan

**Keywords:** Beta thalassemia, Mutations, District Charsadda, Pre-natal diagnosis

## Abstract

**Objective:**

Thalassemia is blood related disease which arises from the reduced level of hemoglobin in red blood cells (RBC), a protein responsible for carrying oxygen inside the body. Considering its widespread occurrence in developing countries like Pakistan, this study aims to investigate the common molecular anomalies of the beta thalassemia disease in district Charsadda, Khyber Pakhtunkhwa.

**Methods:**

This work was done at Abdul Wali Khan University (AWKU) Mardan, Khyber Pakhtunkhwa, Pakistan. The work was performed on the blood samples collected from the patients and their families with beta thalassemia major (n = 13 families) belonged to District Charsadda. The collected blood samples were analyzed for presence of six known mutations with the help of polymerase cha in reaction technique i.e. amplification of refractory mutation system.

**Results:**

Our Study reports six known mutations (IVS-1-5, FSC 8/9, CD 41/42, IVS-1-1, CD 15 and FSC-5) accounting for about 90% of total beta thalassemia genes in this country. Among the reported mutations, IVS 1-5 was the most prevalent beta thalassemia gene in patients belonging to District Charsadda.

**Conclusion:**

The results and findings of the current study may help in accessing the frequency of these common mutations and in initiating pre-natal diagnosis programme in Pakistan.

## INTRODUCTION

Thalassemia represents a group of inherited diseases of hemoglobin which has been demonstrated from the United States and then Italy in 1925. The word “thalassemia”, is a Greek word means “sea blood”. Disorders of hemoglobin are believed to be the most prevalent single-gene disorders. It was believed earlier that these disorders were not present everywhere but restricted to the Mediterranean regions only. It was confirmed later on that the disease is very common and can be found in most parts of the world.^1^-^4^ Among the known disorders, thalassemia is the first to have been studied on molecular level and have given sufficient knowledge about the range of mutation^5^, ^6^ that triggers genetic diseases in humans. This has increased our understanding about the symptoms and other features of the disease which can be used to prevent its occurrence and find ways to treat it after it has been caused. One way to reduce its occurrence in the developing countries is to improve the socioeconomic conditions of the people as it can cause serious public health problem.

B-Thalassemia can be caused whenever there is reduced and defective synthesis of β globin chains.^7^, ^8^ In humans, different types of hemoglobin are found at different developmental stages such as HbA (major hemoglobin) can be normally seen in normal human. It is called major hemoglobin as it accounts for about 90% of the total hemoglobin in the body. In addition to HbA, HbA2 (minor) hemoglobin has been reported from normal adults and comprises for 2-3% of the total hemoglobin. In fetus, HbF is believed to be the major hemoglobin^9^ which can even be found in normal adults but in very trace amounts. In embryonic stages, three different hemoglobin can be observed which are tetramers of two different pairs of globin chains.^10^

It is also worth mentioning that in grown up people and fetus, hemoglobins have chains linked with b, d or g chains. The Z-chains and e-chains can be observed in the embryo. Each distinct globin chain has hemetraces which are aimed to bind oxygen.^11^ The “a” and “b” type of thalassemia is normally being caused due to any mutation/defects in these chains during synthesis. There is also a possibility that defects can occur during synthesis of “d” and “b” chains, or “e”, “g”, “d” and g-chains called db or eg db thalassemia. In rare cases, thalassemia genes can be segregated exactly as segregation of mendelian recessive genes occur. When this disorder is in homozygous form, it is more severe and known as thalassemia major. The term “carrier” refer to conditions when only one of the defective globin gene is inherited.

## METHODS

The blood samples from the targeted patients with beta thalassemia major (n = 400) were taken in ethylenediaminetetraacetic acid (EDTA). The patients included in this study were all transfusion dependent and had been diagnosed earlier as beta thalassemia major with the help of basic haematological parameters, peripheral blood morphology and hemoglobin electrophoresis.

Among the 400 beta thalassemia major patients studied, males and females were 250 and 150 respectively with a mean age of 13.5 years. The collected samples were checked using modified amplification of refractory mutation system (ARMS) for the five common mutations.

In our study, five mutant primers named IVSI-1 (G→T), IVSI-5 (G→C), Del 619, Fr 41-42(- TTCT) and Fr 8-9 (+G) were used. Extraction of DNA from the whole blood was performed according to the manufacturer’s instructions given in Gentra Pure Gene kit (Minneapolis, Minnesota, USA). The ARMS technique was used for characterization and screening of the mutations. For each sample, polymerase chain reaction (PCR) was carried out in two separate tubes, one for normal and other for mutant gene. To check whether PCR conditions are fully optimized, amplification of each sample DNA with control primers A and B were carried out. The expected size of amplicon for the control primers was 861bp.

PCR was performed in 20μl reaction volume composed of 0.5µg template DNA, 10 picomolar of each of the primers used, 2.5UTaq DNA polymerase and 0.2 mM of each of the dNT Pina solution consisting of 10 mM Tris-HC1, 50mM KCl and 1.2mM MgCl2, (Promega). The PCR composed of 25 cycles, each cycle was set as follow:

Denaturation of template DNA using 94°C temperature for 1min, primer annealing at 65°C temperature for 1min and extension at 72°C for 1.5 minutes, final extension at 72°C for 12 minutes.10µl of the amplified PCR product mixed with 3μL of a loading buffer was run on 2% agarose gel stained with ethidium bromide. For electrophoresis, 100 Volts for 50 minutes was used for separation of targeted amplicons. 100bp or 50bp DNA were used as size ladders for estimating the size of amplicon. The amplified PCR product was visualized under ultraviolet (UV) light and pictures taken using a digital camera.

## RESULTS

In the current study, 52 β-thalassemia chromosomes were analyzed to determine its mutations frequency in the residents of District Charsadda. To screen known β-thalassemia causative mutation PCR based tests were performed. Blood samples of 13 transfusion dependent β-thalassemia patients and their family members collected provided useful data for molecular analysis.

Six different β-thalassemia mutations have been identified in Pakistan till date. Previous studies have reported the detailed geographical distribution and allele frequency of these mutations in Pakistan. Two of these common β-thalassemia mutations were found in the present study (Figure 1a and b).

**Fig.1a:**
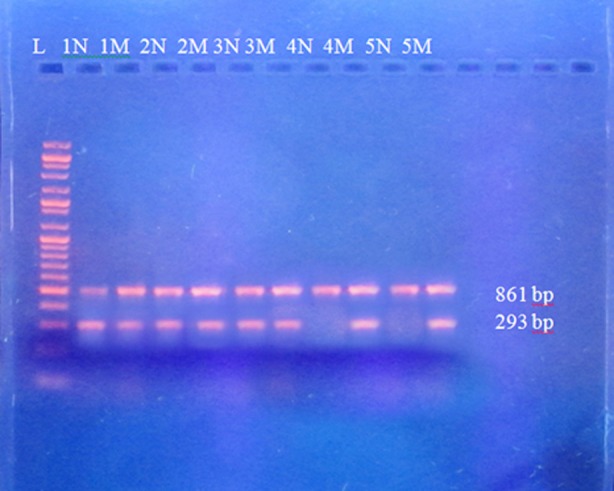
Gel electrophoresis of amplified PCR product for IVS-1-5 mutation. Control primers producing 861bp fragments used as control, common C (forward primer) and IVS-1-5 Mt (mutant) or N (normal) used as reverse primer amplifies PCR product of 293 bp. In this picture “M” indicates mutant products, “N” show normal PCR products. “L” represent 1kb DNA ladder. The father DNA is run in Lane 1N and 1M, it represents that father is heterozygous. The mother DNA is run in Lane 2N and 2M it represents that mother is heterozygous. The 1st child DNA is run in Lane 3N and 3M it represents that the 1^st^ child is heterozygous. The 2nd child DNA is run in Lane 4N and 4M it represents that 2^nd^ child is homozygous. The 3rd child DNA is run in Lane 5N and 5M it represents that the 3rd is homozygous.

**Fig.1b:**
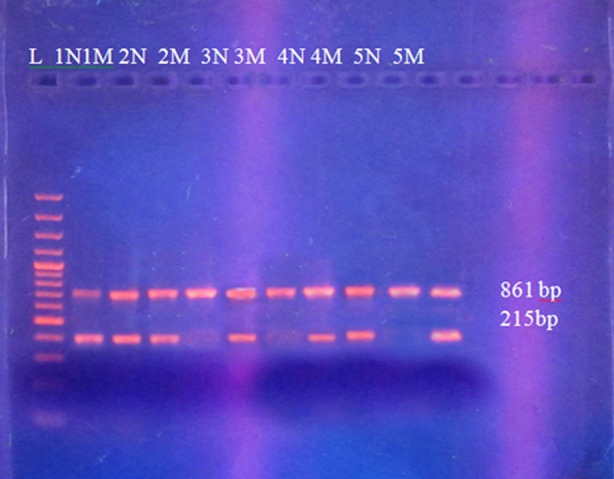
Amplified PCR product of the FSC 8-9 mutation gene. Control primers producing 861bp PCR fragment used as control, common C (forward primer) and FSC 8-9 Mt (mutant) or N (normal) used as reverse primer amplifies 293bp product. On the gel “M” indicates mutant products and “N” show normal PCR products. “L” represents 1kb DNA ladder. The father DNA is run in Lane 1N and 1M it represents that he is heterozygous. The mother DNA is run in Lane 2N and 2M it represents that she is negative for this mutation. The 1st child DNA is run in Lane 3N and 3M it represents that the 1st child is negative for this mutation. The 2nd child DNA is run in Lane 4N and 4M it represents that the 2^nd^ child is heterozygous. The 3rd child DNA is run in Lane 5N and 5M it represents that the 3^rd^ child is homozygous.

PCR employed for the discovery of mutations leading to β-thalassemia in District Charsadda have shown two common mutations i.e. IVS-1-5 and FSC-8/9 (indicated by arrow in Fig. 1a and 1b). Samples amplified with normal primers (N Primers) and mutant primers (Mt Primers) were visualized on the gel.

Some samples were found to have been amplified with mutant primers, they are likely homozygous for the tested mutation. Similarly those samples which managed to amplify with normal primers are presumably negative for the mutation tested. Those samples which amplify with both normal and mutant primers are believed to be heterozygous (carrier) for that mutation.

In PCR, addition of control helped in predicting the expected size amplicon and best interpretation of the results obtained in this study. PCR with vague results was repeated several times until getting clear and demonstrable results. This study reports the molecular analysis of 52 chromosomes using polymerase chain reaction and the presence of common β-thalassemia mutation IVS-1-5 and FSC-8/9. Among the studied 52 chromosomes, molecular defects were found in 13 of the β-thalassemia chromosomes.

## DISCUSSION

The high frequency of β-thalassemia in Pakistani inhabitants is due to several factors including marriages between close relatives, exposure of humans to mutagens and increased birth rate. High frequency of β-thalassemia is one of the reasons for declaring Pakistan among the highest transfusion dependent children.^12^-^14^ According to WHO report^15^, the allele frequency in Pakistan is about 5% and the percentage of individual’s carriers in couples having the risk of this disorder is 25%. About 400 β-thalassemia mutation or molecular defects have been detected worldwide but only 19 β-thalassemia disorders have been revealed in Pakistan.^16^ As its complete treatment has not been discovered yet, the available treatment strategies include iron chelation therapy remedy, transfusion of blood and bone marrow transparent. Iron chelation therapy and transfusion of thalassemia free blood to all patients is difficult due to its high cost and reduced medical facilities.^17^ High treatment cost is one of the reasons that we have failed to control this disorder in our country as yet.

Significant success has been achieved in controlling the spread of β-thalassemia in developed countries by controlling the birth rate from the infected or carrier parents. In these countries, screening of people before marriages^18^ and also of pregnant women is regularly done for the presence of signs of βthalassemia.^19^-^21^ If any symptoms relating to this disease is observed in pregnant women, detailed investigation of the both women and her husband and / or their parents is done and then decided whether the fetus is worth abortion or could be retained without getting effects of thalassemia.

However, such practices cannot be implemented in developing countries like Pakistan due to limited allocated funds towards health and large number of population i.e. more than 150 million population. In addition, lack of awareness is another persistent problem which has resulted in increased number of thalassemia cases in this country. That is the reason most of Children are born with this disease and reduces the chances to control it before birth.^22^ A new strategy developed for proper screening of the infected or doubtful families include index case or retrospective inductive screening^23^ which is economical, feasible and more practicable than other screening methods in Pakistan. In the current study, we have screened the representative population from District Charsadda. This study reports six β thalassemia causative mutations (i.e. IVS-1-5, FSC 8/9, CD 41/42, IVS-1-1, CD 15, FSC-5) and deletion of 619bp mutation in β globin gene identified by polymerase chain reaction.

In our study, out of 13 unrelated transfusion dependent patients, 10 carried IVS-1-5 and 3 carried FSC 8/9 mutation. Our results of getting two common mutations are in agreement with the results of Ahmad et al.^17^ and Khan et al.^16^ This study reinforce that IVS-1-5 is the common β-globin gene mutation/alteration that underlie β-thalassemia in Pakistan. While analyzing two extended families, each with an index case of β-thalassemia, 73% were detected as carriers having 25% risk of producing an affected child if married to a carrier. In these families,25% were identified as carriers. These results show higher carrier frequency (73%) as compared to previous (31%) reports.^17^

It is worth mentioning that the common mutations detected in our study in Pakistan is a subset of total mutations identified in β globin gene throughout the world. Our study illustrates that 94% of mutations causing β-thalassemia in Charsadda regions has been evolved from seven mutations. This study intensifies the need for conducting index case screening of large families rather than general population screening to prevent the spread of β-thalassemia in developing countries like Pakistan.
